# Examining Multimodal AI Resources in Medical Education: The Role of Immersion, Motivation, and Fidelity in AI Narrative Learning

**DOI:** 10.2196/72190

**Published:** 2025-03-18

**Authors:** Chris Jacobs

**Affiliations:** 1 Department of Psychology, University of Bath, Claverton Down, Bath, BA2 7AY, United Kingdom, 44 (0)1225 388388

**Keywords:** artificial intelligence, cinematic clinical narrative, cinemeducation, medical education, narrative learning, AI, medical students, preclinical education, long-term retention, pharmacology, AI tools, GPT-4, image, applicability, CCN

I read with great interest the recent study by Bland [1], “Enhancing Medical Student Engagement Through Cinematic Clinical Narratives: Multimodal Generative AI–Based Mixed Methods Study,” which explored the use of cinematic clinical narratives (CCNs) in medical education. The findings highlighted the potential of multimodal generative artificial intelligence (AI) to enhance engagement and knowledge retention among medical students. While the study effectively demonstrated novel use of AI to modernize case learning, further exploration of engagement mechanisms and broader learning theories may deepen our understanding of how these approaches can be optimized for educational impact.

Engagement in learning is multifaceted and can be linked to immersion [2] and intrinsic motivation [3]. As Bland observed, students exhibited heightened situational interest in CCNs, reinforcing the idea that immersive learning environments can enhance attention and recall. However, beyond the constructivist learning theory discussed in the study, additional models could be considered to expand the theoretical framework for understanding these results. One such model is the Cognitive Affective Model of Immersive Learning framework [4], which emphasizes the interplay between representational fidelity, cognitive load, and technological mediation in shaping learner experiences. Exploring the Cognitive Affective Model of Immersive Learning and similar frameworks could provide a more nuanced perspective on how technology interacts with learner motivation and engagement.

Another area for further inquiry is the role of pretest and posttest methodologies in evaluating learning outcomes. As the study rightly acknowledges, medical education research is often limited to single posttest designs [5], which may not capture students’ initial levels of engagement or attitudes toward learning. Additionally, fidelity of experience is important for recall, as realistic and contextually accurate learning environments can enhance memory retention and application. Implementing pretest assessments could help quantify shifts in engagement, allowing researchers to distinguish between students who are inherently motivated and those whose interest is primarily triggered by the intervention itself. Such an approach could offer valuable insights into how CCNs influence different learner profiles.

Moreover, the conditions under which learning occurs significantly affect student engagement. The study employed a classroom-based intervention, which effectively bridged the gap between controlled laboratory settings and real-world educational environments. Future research might explore how CCNs perform across varied instructional contexts, including self-paced online learning and clinical simulation settings, to assess their adaptability and impact on different learning situations and scenarios.

Bland provides good evidence that multimodal AI resources hold promise in medical education, underscoring the need to adapt to the evolving preferences of modern medical students who increasingly turn to social media for information and engagement. Expanding this research to integrate additional learning theories and measures, with varied instructional settings will help establish best practices for how to use these innovative tools. I appreciate the author’s novel approach and am eager to see how future developments in this field will shape medical education.

## Abbreviations

AI: artificial intelligence
CCN: cinematic clinical narrative
